# Folate-mediated inflammatory microenvironment-responsive nanocarriers for the delivery of Moringa A to target NLRP3 for the treatment of viral pneumonia

**DOI:** 10.1016/j.ijpx.2026.100565

**Published:** 2026-05-07

**Authors:** Taoyuan Zeng, Jiayu Li, Wenyi Yao, Xu Cheng, Dandan Yang, Chunmei Lv, Yongai Xiong

**Affiliations:** Guizhou Provincial Key Laboratory of Innovation and Manufacturing for Pharmaceuticals and School of Pharmacy, Zunyi Medical University, Zunyi, Guizhou 563000, China; Key Laboratory of Basic Pharmacology of Guizhou Province and School of Pharmacy, Zunyi Medical University, Zunyi, Guizhou 563000, China

**Keywords:** Viral Pneumonia, Moringa A, NLRP3, Pyroptosis

## Abstract

Cytokine storm triggered by respiratory viral infection is the core pathogenic mechanism of severe pneumonia, and excessive activation of the NLRP3 inflammasome is a critical link in inducing this storm and subsequent lung tissue damage. This study targets the NLRP3 inflammasome to investigate the therapeutic effects, mechanisms of action, and targeted delivery advantages of folic acid-modified nanoparticles encapsulating Moringa A (MA NPs) for viral pneumonia. Utilizing techniques such as cellular experiments, mouse model validation, molecular docking, bio-layer interferometry (BLI), immunohistochemistry, immunofluorescence, and histopathology, this study systematically analyzed the effects of MA NPs on the NLRP3 inflammasome pathway, pyroptosis, macrophage polarization, and lung tissue injury, while also validating the targeting efficacy of the folic acid-modified nanodelivery system. The results showed that MA NPs significantly reduced the expression of NLRP3, ASC, Caspase-1, and GSDMD in H1N1 virus-infected cells, decreased the levels of pyroptosis-related cytokines, and MA was confirmed to be an NLRP3 inhibitor. In mouse models, MA NPs reduced the lung index, downregulated the expression of pro-inflammatory cytokines in lung tissue, and alleviated pathological and ultrastructural damage to lung tissue. Furthermore, MA NPs inhibited the excessive activation of the NLRP3 inflammasome and promoted the polarization of macrophages from the M1 phenotype to the M2 phenotype, thereby alleviating the pulmonary inflammatory microenvironment. Therefore, MA NPs can repair lung tissue damage by inhibiting excessive activation of the NLRP3 inflammasome and regulating macrophage polarization, and combined with the folic acid-targeted delivery system, achieve precision treatment for viral pneumonia. This provides a new approach and experimental basis for the synergistic intervention of viral pneumonia.

## Graphical Abstract

**Figure:**
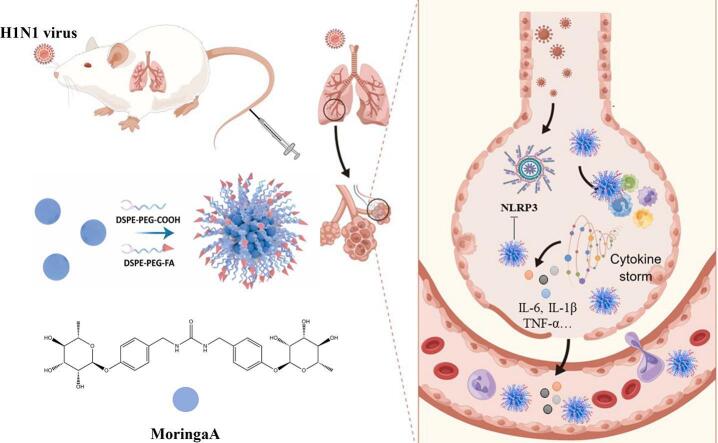

## 1 Introduction

In recent years, the continuous emergence of novel viral variants has posed severe challenges to the prevention and treatment of viral pneumonia, with its pathological impact extending beyond local lung injury to exhibit multi-system and long-term effects. Emerging evidence indicates that viral pneumonia not only manifests as typical respiratory symptoms such as fever, cough, and dyspnea but can also progress to acute respiratory distress syndrome (ARDS) in severe cases, posing life-threatening risks, particularly in infants, the elderly, and immunocompromised populations(Cilloniz et al., 2024; Grune et al., 2024). More notably, severe viral pneumonia leaves persistent immune scars in the lungs, reshaping the pulmonary immune microenvironment through epigenetic reprogramming and sustaining a state of chronic inflammation(Baker et al., 2024; Dukhinova et al., 2021). This significantly increases the long-term risk of lung cancer, with studies showing that even patients without pre-existing conditions face a 1.24-fold increased incidence of lung cancer following severe infection. Furthermore, certain viruses, such as SARS-CoV-2, can induce extrapulmonary injuries. Studies in infected mouse models have confirmed the induction of neuroinflammation, accelerating the pathological progression of associated neurodegenerative diseases, thereby broadening the spectrum of its detrimental effects(Rodrigues et al., 2025). Conventional treatment strategies primarily rely on supportive care and antiviral agents; yet these approaches are often limited by poor bioavailability; inadequate targeting; and systemic side effects; hindering effective modulation of the inflammatory microenvironment and prevention of disease progression and long-term sequelae(Zhang et al., 2025a).

Folate (FA)-mediated inflammatory microenvironment-responsive nanocarriers have emerged as a prominent research focus in nanomedicine, offering a promising paradigm for precision therapy of viral pneumonia. The inflammatory microenvironment, a hallmark of viral pneumonia pathogenesis, is characterized by excessive reactive oxygen species (ROS) accumulation, decreased pH, aberrant activation of inflammatory cells, and elevated expression of pro-inflammatory cytokines, providing intrinsic targets for the rational design of responsive nanocarriers(Chang et al., 2024; Wu et al., 2026). During viral pneumonia, folate receptor (FR) is highly and specifically overexpressed on the surface of activated macrophages(Song et al., 2022; Zheng et al., 2025), alveolar epithelial cells, and other inflammatory lung cells, whereas its expression remains low in normal lung tissues and healthy cells. This differential expression pattern enables folic acid (FA)-mediated active targeting to inflammatory lung lesions, facilitating precise drug accumulation at damaged pulmonary sites. As a targeting ligand, FA exhibits several ideal properties, including low immunogenicity, excellent biocompatibility, stable chemical structure, ease of modification on nanoparticle surfaces, and low production cost—features that confer good biosafety and translational potential to the nanosystem. Notably, the significant upregulation of FRs on inflammatory cells (e.g., activated macrophages) and within lesioned tissues during viral pneumonia provides a molecular basis for precise nanocarrier delivery, thereby effectively overcoming key limitations of conventional therapeutics, such as poor retention at pathological sites and extensive distribution in non-target tissues(Ebrahimnejad et al., 2022; Kesharwani et al., 2024).

These intelligent nanocarriers enable a cascade effect of targeted accumulation – microenvironmental responsiveness - controlled drug release. Folate-mediated active targeting facilitates efficient accumulation of the nanocarriers within pulmonary inflammatory lesions, minimizing distribution in healthy tissues and consequently reducing systemic toxicity(Wang et al., 2022). Upon reaching the inflammatory microenvironment; the nanocarriers sense pathological cues such as elevated ROS levels and acidic pH; triggering structural dissociation and achieving controlled; sustained drug release at the target site(Liu et al., 2024; Yang et al., 2024). This responsive release mechanism prevents premature drug degradation prior to target engagement, markedly enhancing local drug concentration and bioavailability. Furthermore, the versatile nanocarrier platform can be co-loaded with antiviral agents, anti-inflammatory cytokines, antioxidants, and other therapeutic modalities, enabling synergistic antiviral and anti-inflammatory effects alongside pulmonary injury repair. This multifaceted approach directly inhibits viral replication while concurrently scavenging excessive ROS, modulating macrophage polarization, and suppressing pro-inflammatory signaling cascades. Consequently, this strategy orchestrates the remodeling of the pulmonary inflammatory microenvironment, disrupting the deleterious cycle of persistent inflammation - tissue damage - long-term complications(Chang et al., 2024; Lv et al., 2024b; Muhammad et al., 2022). It also holds potential to mitigate post-severe infection immune scars and reduce the long-term risk of malignancy. Recent in vivo studies have validated that folate-functionalized responsive nanocarriers effectively target inflamed tissues, significantly enhancing therapeutic efficacy and improving disease prognosis, laying a robust foundation for their clinical translation(Li et al., 2025b). This innovative strategy holds promise as a paradigm shift, overcoming the limitations of conventional therapies and improving both short-term outcomes and long-term prognosis for patients with viral pneumonia.

Moringa A (MA), a novel compound with 99.8% purity, was previously isolated and structurally identified from *Moringa oleifera* seeds in our laboratory. Subsequent pharmacological validation confirmed that MA exerts prominent anti-influenza viral activity and strong anti-inflammatory effects in both in vitro and in vivo experimental models(Lv et al., 2024a; Wu et al., 2025; Xiong et al., 2021; Xiong et al., 2022). These findings underscore its potential as a promising lead compound for antiviral and anti-inflammatory drug development. However, physicochemical characterization has revealed that MA possesses poor water solubility and pronounced hydrophobicity. Such properties typically result in suboptimal absorption and low bioavailability in vivo, hindering the attainment of effective therapeutic concentrations within target tissues or organs. This limitation restricts the full realization of its pharmacological efficacy and impedes further pharmacological and pharmacodynamic evaluation. Therefore, building upon the established potent activity of MA, further in-depth investigations aimed at improving its solubility and bioavailability are urgently required. Strategies such as structural modification or formulation optimization (e.g., nanoparticle-based delivery systems, cyclodextrin inclusion complexes) are essential to maximally exploit its pharmaceutical potential and lay the groundwork for subsequent anti-influenza drug development.

NLRP3 has been demonstrated to play a critical role in the progression of viral pneumonia, and inhibiting NLRP3 is essential for the control of viral pneumonia. Currently, a growing number of nanotherapies targeting the NLRP3 inflammasome have been developed to ameliorate excessive inflammatory damage during viral pneumonia, yet several critical limitations still restrict their in vivo application and translational value. Most reported NLRP3-targeted nanoplatforms merely exert a single anti-inflammatory effect without directly suppressing viral replication, making it difficult to block disease progression at the initial infection stage. Moreover, the therapeutic agents commonly used are synthetic small-molecule NLRP3 inhibitors, which often suffer from inevitable cytotoxicity, poor biocompatibility, and potential side effects. Conventional nano-delivery systems further rely on passive targeting via the enhanced permeability and retention effect or inflammatory microenvironment response, leading to insufficient lung accumulation, nonspecific tissue distribution, and weakened therapeutic specificity. Consequently, these monomodal strategies cannot simultaneously resolve viral infection and the secondary inflammatory storm, thus hardly achieving satisfactory comprehensive efficacy against viral pneumonia.

In this study, leveraging the pathophysiological characteristics of the inflammatory microenvironment in viral pneumonia, we designed and constructed a folate (FA)-functionalized, inflammatory microenvironment-responsive nano-delivery system. This system utilizes amphiphilic block copolymers, DSPE-PEG-COOH and DSPE-PEG-FA, as co-assembling nanocarriers for the encapsulation of MA. Within this system, the folate ligand conjugated to DSPE-PEG-FA specifically recognizes folate receptor-β (FR-β), which is highly expressed on the surface of activated macrophages at sites of inflammation, thereby conferring active targeting capability to the nanoparticles. Concurrently, the DSPE-PEG-COOH component responds to the weakly acidic pH (approximately 6.5) characteristic of the inflammatory microenvironment, enabling intelligent, triggered drug release. Utilizing this nano-delivery system, MA can be precisely delivered to pulmonary inflammatory lesions and efficiently internalized by target cells, thereby maximizing its therapeutic efficacy against viral pneumonia. Furthermore, through a series of in vitro and in vivo experiments, this study aims to preliminarily investigate the regulatory effects of this nano-formulated drug on the NLRP3 inflammasome signaling pathway and elucidate the underlying molecular mechanisms involved in its therapeutic action against viral pneumonia. The outcomes of this research are anticipated to provide a novel strategy and experimental basis for the development of innovative therapeutic interventions for viral pneumonia.

## 2 Materials and methods

### 2.1 Materials

DSPE-PEG_1k_-COOH (Cat. No. 80031301–3400) was purchased from Tanshtech; DSPE-PEG_1k_-FA (Cat. No. PS2-E1FA-5 K) was purchased from Shanghai Ponsure Biotechnology Co., Ltd.; the ultrafiltration centrifuge tube (Cat. No. UFC-150-030-PES) was purchased from Beijing Lanjieke Technology Co., Ltd.; Cy5.5 (Batch No. 154528) was purchased from Anhui Zesheng Technology Co., Ltd.; DMEM/F12 medium (Cat. No. L310KJ) was purchased from Shanghai Yuanpei Biotechnology Co., Ltd.; fetal bovine serum (Cat. No. 10099-141C) was purchased from Gibco, USA; Coumarin 6 (Batch No. RH643784) was purchased from Guangzhou Ron Biotechnology Co., Ltd.; 4% paraformaldehyde fixative solution (Cat. No. BL539A) was purchased from Beijing Biosharp Company. MCC950(NLRP3 inhibitor, Cat. No. T3701) was purchased from TargetMol Chemicals Inc., Antifade Mounting Medium with DAPI (Cat. No. S2110) and Lipopolysaccharide (Cat. No. L8880) were both purchased from Beijing Solarbio Technology Co., Ltd.; HRP-conjugated Goat Anti-Rabbit IgG (H + L) (Cat. No. SA00001–2) was purchased from Wuhan Sanying Biotechnology Co., Ltd.; iFluor™ 488 Conjugated Goat anti-rabbit IgG polyclonal Antibody (Cat. No. SA00001–2) was purchased from Hangzhou HuaAn Biotechnology Co., Ltd.; GSDMD antibody (Cat. No. 20770–1-AP), NLRP3 antibody (Cat. No. 30109–1-AP), Caspase-1 antibody (Cat. No. 31020–1-AP), and Beta Actin (Cat. No. 66009–1-Ig) were all purchased from Wuhan Sanying Biotechnology Co., Ltd.; ASC-1 antibody (Cat. No. ER62748) was purchased from Hangzhou HuaAn Biotechnology Co., Ltd.; the Mouse Interleukin-6 (IL-6), Interleukin-18 (IL-18), Tumor Necrosis Factor α (TNF-α), Interleukin-1β (IL-1β), and Lactate Dehydrogenase (LDH) ELISA kits (Cat. Nos. JM-02446 M1, JM-02452 M1, JM-02415 M1, JM-02323 M1, and JM-11330 M1, respectively) were all purchased from Jiangsu Jingmei Biotechnology Co., Ltd.

### 2.2 Virus and cell lines

Influenza A virus A/Puerto Rico/8/34, obtained from the American Type Culture Collection (ATCC). The mouse lung epithelial cell line MLE-12 was purchased from Shanghai Binsui Biotechnology Co., Ltd.

### 2.3 Experimental animals

A total of 60 healthy SPF-grade KM mice, half male and half female, aged 6 weeks and weighing 18–22 g, were provided by Hunan SJA Laboratory Animal Co., Ltd. All mice were housed in cages (5 per cage) in an SPF-grade animal facility under a 12 h light/12 h dark cycle, with the ambient temperature controlled at 22 ± 2 °C and relative humidity maintained at 50%–60%. The animals had free access to standard maintenance diet and sterilized water. All experiments were conducted after one week of acclimatization feeding. All animal studies were approved by the Ethics Committee of Zunyi Medical University (Permit No. ZMU21–2503-287) and performed in accordance with the Laboratory Animal Welfare and Ethics Committee of China.

## 3 Methods

### 3.1 Preparation and purification of MA-loaded nanoparticles (MA NPs)

5 mg of DSPE-PEG-COOH (A) and 5 mg of DSPE-PEG-FA (B) were each dissolved in methanol to prepare solutions with a concentration of 2.5 mg/mL. The two material solutions were mixed according to a volume ratio of A to B of 7:3 to obtain a mixed solution (AB). MA was weighed according to a mass ratio of AB to MA of 3:1, and then added to the AB mixed solution. The mixture was stirred to dissolve, resulting in a drug-containing mixed solution. This mixed solution was slowly and uniformly added dropwise to purified water under magnetic stirring. Stirring continued for 3 h. Methanol was removed by rotary evaporation, yielding a nanoparticle solution loaded with MA. Using a 30 KDa ultrafiltration centrifuge tube, free MA was removed by centrifugation at 2500 rpm. The nanoparticles were washed three times with deionized water. Finally, the appearance, morphology, particle size, polydispersity index (PDI), etc., of the nanoparticles were measured.

### 3.2 Stability test of MA NPs

The samples were stored at room temperature (25 °C) and 4 °C for 30 days, respectively. Changes in the particle size and polydispersity index (PDI) of the nanoparticles were monitored using a laser particle size analyzer on days 0, 1, 3, 5, 7, 12, 15, 20, 25, and 30.

### 3.3 Determination of cumulative release rate of MA NPs

Fifteen milligrams of freeze-dried nanoparticles (equivalent to 3.0195 mg of MA) were weighed and dispersed in 5 mL of PBS. One milliliter of this dispersion was placed into a dialysis bag (molecular weight cutoff: 3500 Da). Both ends of the bag were sealed, and it was immersed in 30 mL of PBS release media with different pH values. Dialysis was performed in a constant temperature shaker at 37 °C and 110 r/min. A free MA group was set up as a control. At predetermined time points (0.17, 0.5, 0.75, 1, 2, 4, 6, 8, 10, 12, 24, 48, and 72 h), 1 mL of the dialysis external solution was collected, and an equal volume of fresh PBS was immediately added to maintain the volume. The collected samples were filtered through a 0.22 μm membrane and analyzed under the aforementioned chromatographic conditions. Based on the measured drug concentrations, the cumulative release rate at each time point was calculated, and release curves were plotted to evaluate the in vitro drug release behavior of the nanoparticles under different pH conditions.

### 3.4 Cellular uptake

Using Coumarin-6(C-6) as a fluorescent probe, the uptake efficiency of the delivery materials by MLE-12 cells and H1N1 virus-infected MLE-12 cells was investigated. Cells were seeded at a density of 5 × 10^4^ cells per well in 500 μL of medium into a 24-well plate containing pre-placed cell slides. The plate was incubated in a 37 °C, 5% CO₂ incubator for 24 h to allow complete cell adhesion. The plate was then removed, the culture medium was discarded, and the cells were washed twice with PBS. The preparation method for Coumarin-6-loaded nanoparticles (C-6 NPs) was the same as that used for MA NPs.The prepared C-6 NPs were added, along with setting up a blank control group and a free C-6 group. Each well received 500 μL of the respective treatment, with three replicate wells per group. The plate was incubated in a 37 °C, 5% CO₂ incubator protected from light for 1, 2, and 4 h. After incubation, the plate was removed, and the cells were washed three times with PBS. The cells were then fixed with 200 μL/well of 4% paraformaldehyde for 20 min, followed by three washes with PBS. The cell slides were inverted and mounted onto glass slides pre-dropped with an anti-fluorescence quencher containing DAPI. After sealing, the slides were placed in the dark for 10 min and then observed under a confocal microscope.

### 3.5 Hemolysis assay of MA NPs

For the hemolysis assay, a 2% red blood cell suspension was first prepared by collecting fresh blood from healthy mice into anticoagulant tubes. Under a clean bench, PBS was added to the blood, gently mixed, and centrifuged at 10,000 r/min for 5 min. The supernatant was discarded, and the pellet was resuspended in fresh PBS and centrifuged again; this washing process was repeated three times until the supernatant was clear and colorless. The obtained erythrocytes were then diluted with an appropriate volume of PBS to achieve a 2% suspension. Subsequently, 950 μL of this suspension was mixed with 50 μL of Triton X-100 (positive control), PBS (negative control), or MA nanoparticle solution, respectively, and incubated at 37 °C for 1, 2, 4, 8, and 12 h. After each incubation, the mixtures were centrifuged at 10,000 r/min for 5 min, and the supernatants were collected and photographed. Finally, the absorbance (OD value) of hemoglobin in the supernatant was measured at 562 nm using a microplate reader with three replicate wells per sample, and the hemolysis rate was calculated accordingly.

### 3.6 In vivo lung tissue targeting test

To assess the lung-targeting efficiency of the nanoparticles, targeted and non-targeted nanoparticles encapsulating the fluorescent dye Cy5.5 were prepared. The targeted nanoparticles were modified with DSPE-PEG-FA (referred to as DSPE-FA@Cy5.5), while the non-targeted counterparts were prepared using DSPE-PEG-COOH without FA (referred to as DSPE-COOH@Cy5.5). Mice were used as the animal model. All mice were randomly divided into three groups: free Cy5.5 dye group (mice were injected with normal saline through the tail), lung-targeted delivery group (mice were injected with DSPE-FA@Cy5.5 through the tail), and non-targeted delivery group (mice were injected with DSPE-COOH@Cy5.5 through the tail). Each group consisted of three mice per time point for three subsequent time points. 48 h before the experiment, the mice were fasted but allowed free access to water. To establish a viral pneumonia model, mice were intranasally infected with ×10 minimum lethal dose (MLD_50_) of H1N1 viruses in a 50-μl volume prior to nanoparticle administration. Subsequently, 0.2 mL of the respective Cy5.5 nanoparticle solution was injected through the tail vein. At 3, 6, and 9 h post-injection, mice from each group were euthanized by cervical dislocation. The heart, liver, spleen, lungs, and kidneys were dissected and collected. Ex vivo fluorescence imaging of the organs was performed using an in vivo imaging system. The biodistribution of the nanoparticles in various organs was evaluated by detecting the fluorescence signal intensity of Cy5.5, thereby verifying their lung-targeting capability.

### 3.7 In vitro efficacy study

#### 3.7.1 Cytotoxicity

MLE-12 cells in good growth condition were seeded into 96-well plates at a density of 5 × 10^4^ cells per well in 100 μL, and cultured at 37 °C with 5% CO₂ until a confluent monolayer formed. The original culture medium was aspirated, and the cells were washed three times with PBS. Then, cell maintenance medium containing serial twofold dilutions of the test compounds was added: free Moringa A group (400, 200, 100, 50, 25, 12.5, 6.25 μM) and MA NPs group (1, 5, 10, 20, 40, 80, 100 μM). Each concentration was added in 100 μL per well, with six replicate wells per group, and a control group was also included. The plates were incubated at 37 °C with 5% CO₂ for 24 h. After incubation, the culture medium was aspirated, the cells were washed three times with PBS, and 90 μL of cell maintenance medium along with 10 μL of CCK-8 reagent were added to each well. Following incubation at 37 °C for 1 h, the absorbance of each well was measured at 450 nm using a microplate reader. The half-toxic concentration (TC₅₀) was calculated using the Reed-Muench method.

#### 3.7.2 Cell grouping and treatment

MLE-12 cells in the logarithmic growth phase were seeded into 96-well plates at a density of 5 × 10^4^ cells per well in 100 μL. According to different treatment protocols, the cells were divided into a control group, a model group, a NLRP3 inhibitor MCC950 group (5 μM),a free MA group (treated with 12.5 μM MA), an MA NPs high-dose group (treated with 10 μM MA NPs), and an MA NPs low-dose group (treated with 5 μM MA NPs), with six replicate wells per group. The cells in each experimental group were incubated at 37 °C with 5% CO₂ for 24 h to allow the formation of a confluent monolayer. The original culture medium was then aspirated, and the cells were washed three times with PBS. To induce infection, all groups except control were exposed to H1N1 at a MOI of 5 for 24 h, while control and model were given blank medium. Following this incubation, blank medium or drugs were added to cells according to their respective groups, and the treatment was terminated after 48 h of incubation, with daily monitoring of cytopathic effects (CPEs).

#### 3.7.3 Elisa assay

After treatment, the cell supernatant from each experimental group was collected. The levels of IL-6, IL-18, IL-1β, TNF-α and LDH in the supernatant were detected following the instructions of the ELISA kit.

#### 3.7.4 Cellular transcriptomics analysis

To further investigate the therapeutic mechanism of MA in viral pneumonia, after completing the in vitro pharmacodynamic evaluation, we employed transcriptome sequencing technology based on the Illumina platform to systematically analyze the effect of MA on the gene expression in H1N1 virus infected cells, aiming to screen key differentially expressed genes and elucidate their regulatory networks. The experiments were commissioned to Shanghai Bioprofile Biotechnology Co., Ltd., and the specific procedures were as follows: First, mRNA with polyA tails was enriched from total RNA using Oligo(dT) magnetic beads, and then fragmented to approximately 300 bp by ion fragmentation to optimize the sequencing structure. Subsequently, random hexamer primers were used for reverse transcription to synthesize the first-strand cDNA, followed by the construction of double-stranded cDNA. After PCR amplification, library fragments with an average length of approximately 450 bp were screened and purified. The library was quality-checked using an Agilent 2100 Bioanalyzer, after which the effective concentration was determined, and libraries with different Index labels were mixed in proportion. Finally, single-stranded libraries were obtained through dilution and denaturation, and paired-end sequencing was performed on an Illumina sequencer to obtain comprehensive and reliable transcriptome data for subsequent mechanistic analysis.

#### 3.7.5 Western blotting

After treatment, the culture medium was discarded, and the cells were washed three times with pre-chilled PBS. RIPA lysis buffer and protease inhibitors freshly prepared at a ratio of 100:1 were added, and the cells were lysed on ice for 30 min, during which the mixture was pipetted every 10 min to ensure complete lysis. The lysate was collected and centrifuged at 12,000 r/min for 15 min at 4 °C. The supernatant was collected, and the protein concentration was determined using the BCA method. After adding 5× protein loading buffer, the samples were boiled in a water bath for 10 min, aliquoted, and stored at −20 °C for further use. Equal amounts of protein samples were subjected to SDS-PAGE gel electrophoresis and then wet-transferred onto 0.45 μm PVDF membranes at a constant current of 400 mA. After blocking with 5% non-fat milk at room temperature for 1 h, the membranes were incubated with primary antibodies against NLRP3, Caspase-1, and ASC (all diluted in QuickBlock™ Western Primary Antibody Dilution Buffer) overnight at 4 °C. The next day, the membranes were washed three times with TBST, 5 min each, followed by incubation with the corresponding secondary antibodies (diluted 1:4000) at room temperature for 1 h. After washing with TBST, ECL substrate was added for signal development, and images were captured for analysis of protein expression levels.

#### 3.7.6 Immunofluorescence (IF) assay

After drug treatment, the culture medium was aspirated, and the cells were washed three times with PBS. Then, 200 μL of 4% paraformaldehyde was added per well for fixation for 30 min. After fixation, the cells were washed three times with PBS (3 min each wash), and 500 μL of QuickBlock™ Immunostaining Blocking Buffer was added per well for blocking at room temperature for 1 h. Following blocking, the cells were washed three times with PBS (3 min each wash), and 500 μL of primary antibody against GSDMD, diluted at 1:500 in QuickBlock™ Primary Antibody Dilution Buffer for Immunostaining, was added per well and incubated overnight at 4 °C. The primary antibody was then recovered, and the cells were washed three times with PBS (3 min each wash). Subsequently, 500 μL of fluorescent secondary antibody, diluted at 1:1000 in QuickBlock™ Secondary Antibody Dilution Buffer for Immunostaining, was added per well and incubated on a shaker for 1 h in the dark. After the secondary antibody was recovered, the cells were washed five times with PBS (3 min each wash). The cell slides were then inverted onto glass slides pre-dotted with 10 μL of antifade mounting medium containing DAPI. Excess liquid was removed with filter paper, and a small amount of quick-drying nail polish was applied for fixation. Finally, the samples were visualized and imaged using a confocal laser scanning microscope.

#### 3.7.7 Bio-layer interferometry (BLI) assay

The interaction between NLRP3 and MA was detected using a molecular interaction instrument (OCTET-R2, Sartorius Lab Instruments GmbH & Co.). HIS1K sensors were immersed in buffer for 10 min prior to use. NLRP3 protein (HY-P790108, MCE) was diluted to 434 nM with buffer (PBST, 0.05% Tween-20). MA was diluted with PBST to 200 μM and subsequently subjected to two-fold serial dilutions. A 200 μL aliquot of NLRP3 protein solution was loaded onto HIS1K sensors until the signal reached approximately 1. The association time for the small molecule was 150 s, and the dissociation time was 100 s. Experimental results were analyzed using Octet Data Analysis Software 9.0.

### 3.8 In vivo pharmacodynamic study of MA NPs in the treatment of viral pneumonia

Mice were randomly divided into 6 groups according to body weight (10 mice per group): normal control group (Control), model group (Model), NLRP3 inhibitor group (MCC950 group, mice received MCC950 dissolved in PBS(50 mg/kg) via intraperitoneal injection), Free MA group (mice received MA dissolved in PBS via tail vein injection, 2 mg/kg), MA NPs high-dose group (mice received MA NPs solution at a dose of 10 mg/kg via tail vein injection), and MA NPs low-dose group (mice received MA NPs solution at a dose of 5 mg/kg via tail vein injection). Mice in the normal control and model groups received an equal volume of normal saline via tail vein injection. On the first day, all mice except those in the normal control group were intranasally infected with 10× minimum lethal dose (MLD₅₀) of H1N1 virus in a 50-μL volume. The first drug administration was given 48 h post-infection, the second administration was given 72 h post-infection, and the third administration was given 96 h post-infection. Six hours after the third administration, mice in each experimental group were euthanized, and lung tissues were collected for subsequent analysis.

(1) Lung Index Measurement

Before lung tissue collection, mice were weighed. After cervical dislocation, the whole lungs were dissected, weighed, and photographed to record the pathological condition of the lungs. The lung index was calculated as lung index = lung weight / body weight.

(2) Detection of Inflammatory Cytokine Levels in Mouse Lung Tissue

The expression levels of IL-1β, IL-6, IL-18, and TNF-α in lung homogenates were detected according to the instructions of the ELISA kits.

(3) Hematoxylin and Eosin (HE) Staining

Lung tissues were fixed in 4% paraformaldehyde for 12 h, then rinsed with PBS, dehydrated in a graded ethanol series, cleared with xylene, embedded in paraffin, and sectioned. Finally, the sections were stained with HE, dehydrated in a graded ethanol series, mounted, and observed under a microscope.

(4) Transmission Electron Microscopy (TEM)

Fresh mouse lung tissues were fixed in 2.5% glutaraldehyde at 4 °C for 24 h. After rinsing with phosphate buffer, the tissues were post-fixed in 1% osmium tetroxide for 2 h. Subsequently, the tissues were dehydrated in a graded ethanol series (50%, 70%, 90%, and 100%), infiltrated, embedded in epoxy resin, and polymerized. Ultrathin sections (70–90 nm) were cut using an ultramicrotome, double-stained with uranyl acetate and lead citrate, placed on copper grids, and observed under a transmission electron microscope. The analysis focused on typical ultrastructural features of pyroptosis, including cell membrane integrity, cell swelling, plasma membrane pore formation, organelle edema, and chromatin condensation.

(5) Immunohistochemistry (IHC)

Fixed lung tissues were embedded, sectioned, and subjected to immunohistochemical staining as follows: Sections were placed in EDTA antigen retrieval buffer (pH 8.0) and heated at 95 °C for 30 min. After natural cooling, sections were washed three times with PBS (5 min each wash). To block endogenous peroxidase activity, sections were incubated in 3% H₂O₂ solution at room temperature in the dark for 25 min, followed by three PBS washes (5 min each). Subsequently, sections were blocked with 3% BSA at room temperature for 30 min. After blocking, sections were incubated with primary antibodies (NLRP3, 1:500; GSDMD, 1:1000; Caspase1, 1:1000; ASC, 1:1000) in a humidified chamber at 4 °C overnight. After removing the primary antibodies, sections were washed three times with PBS (5 min each) and incubated with secondary antibodies at room temperature for 50 min. Following removal of secondary antibodies, sections were washed three times with PBS (5 min each). Freshly prepared DAB substrate was then applied, and the staining process was monitored under a microscope. When brownish-yellow signals appeared, the reaction was terminated by immersing the sections in water. Sections were then counterstained with hematoxylin for 3 min, differentiated, blued, dehydrated in graded ethanol, cleared in butanol and xylene (5 min each), and mounted with mounting medium. Finally, sections were observed and images were captured under a microscope.

(6) Immunofluorescence (IF)

Fixed lung tissues were embedded, sectioned, and processed for immunofluorescence staining. Antigen retrieval, serum blocking, primary antibody incubation, and secondary antibody incubation were performed sequentially. Primary antibodies used were CD86 (1:500) and CD206 (1:400). Secondary antibodies used were Alexa Fluor 488-conjugated goat anti-rabbit IgG (1:400) and CY3-conjugated goat anti-rabbit IgG (1:300). After secondary antibody incubation, sections were washed three times with PBS (5 min each) and incubated with DAPI staining solution in the dark at room temperature for 10 min for nuclear counterstaining. The DAPI solution was removed, and sections were washed three times with PBS (5 min each). Autofluorescence quenching reagent was applied for 5 min, followed by rinsing under running water for 10 min to quench tissue autofluorescence. Finally, sections were mounted with an anti-fade mounting medium and observed using a confocal laser scanning microscope for image acquisition.

### 3.9 Biosafety evaluation of MA NPs

For the safety evaluation of MA NPs, mice with viral pneumonia were subjected to daily tail vein injection of high-dose MA NPs for two consecutive weeks. After the experiment, the heart, liver, spleen, lung and kidney of mice were dissected and collected for pathological examination, so as to preliminarily evaluate the biosafety of MA NPs during the treatment cycle.

### 3.10 Statistical analysis

All data in this study were processed using SPSS 29.0 software. Data are expressed as mean ± standard deviation (SD). One-way analysis of variance (ANOVA) was used for comparisons among multiple groups. For data with homogeneity of variances, Bonferroni post-hoc test was applied, and the least significant difference (LSD) method was used for intergroup comparisons. A *P*-value <0.05 was considered statistically significant.

## 4 Results

### 4.1 Characterization and stability of MA NPs

**Fig. 1:**
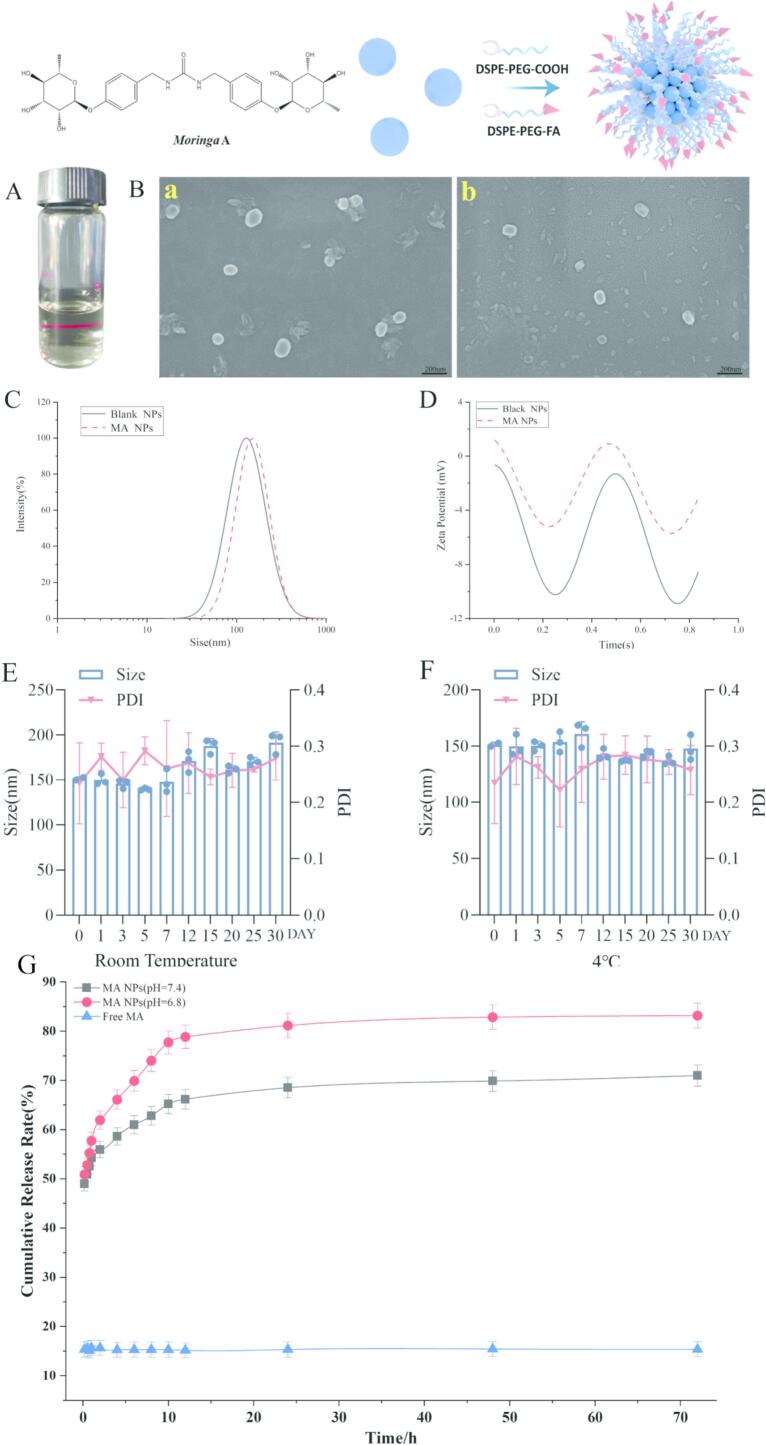
**Preparation and characterization of MA NPs.** A. Tyndall effect of MA NPs solution. B. SEM images of MA NPs (a. MA NPs; b. Blank NPs). C. Particle size distribution. D. Zeta potential distribution. E. Stability of MA NPs under room temperature storage. F. Stability of MA NPs under 4 °C storage. G. Cumulative release of MA NPs in PBS solutions with different pH.

MA NPs presented as a transparent solution without suspended particles (Fig. 1A). SEM analysis of blank NPs and MA NPs (Fig. 1B) revealed that MA NPs exhibited a spherical-like morphology with smooth surfaces, relatively uniform shape, and homogeneous distribution without significant aggregation, indicating good dispersion stability and the formation of structurally intact nanoparticles, with morphological and structural characteristics meeting the requirements for drug delivery systems. The hydrodynamic size and distribution of MA NPs were determined by dynamic light scattering, and surface charge stability was evaluated by zeta potential analysis. As shown in Fig. 1C and D, the average particle size of blank NPs was 134.93 nm, with a zeta potential of −22.3 mV. After drug loading, the particle size of MA NPs increased to 151.43 nm, and the absolute value of zeta potential decreased to −16.32 mV, while still remaining above 10 mV, meeting the size requirements for nanomedicine delivery systems. Fig. 1E shows the changes in particle size and PDI of MA NPs over 30 days at room temperature (25 °C). Under room temperature storage conditions, the particle size of MA NPs remained relatively stable over time, with values fluctuating within a reasonable range at each time point without significant increase or aggregation tendency, indicating good physical stability of the nanostructure during the observation period. Meanwhile, the PDI remained below 0.3, meeting the acceptance criteria. The stability of MA NPs at 4 °C was further evaluated. As shown in Fig. 1F, under this condition, the particle size remained consistently within a reasonable range, and the PDI remained below 0.3 throughout.

Fig. 1G shows the cumulative release profiles of free MA and MA NPs in PBS solution at 37 °C with pH 7.4 or pH 6.8. Free MA exhibited minimal release over 72 h, with only approximately 16% of the drug released. In contrast, MA NPs displayed a rapid release phase within the first 4 h in both pH 7.4 and pH 6.8 PBS, followed by a slower release phase. The cumulative release rate of MA NPs in pH 7.4 PBS reached 71.98% over 72 h, whereas that in pH 6.8 PBS was 83.87%, indicating that MA NPs exhibited greater release in the mildly acidic inflammatory environment.

### 4.2 Specific uptake of nanoparticles by MLE-12 cells

**Fig. 2:**
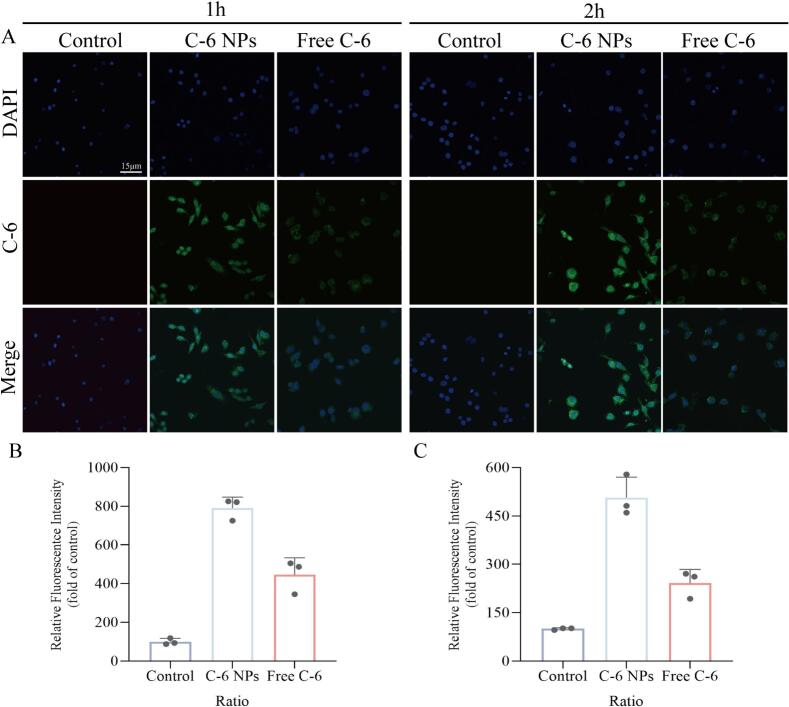
**Uptake of coumarin-6 by normal MLE-12 cells.** A. Fluorescence detection image of coumarin-6 uptake by normal MLE-12 cells; B. Quantitative fluorescence analysis of coumarin-6 in MLE-12 cells at 1 h. C. Quantitative fluorescence analysis of coumarin-6 in MLE-12 cells at 2 h.

**Fig. 3:**
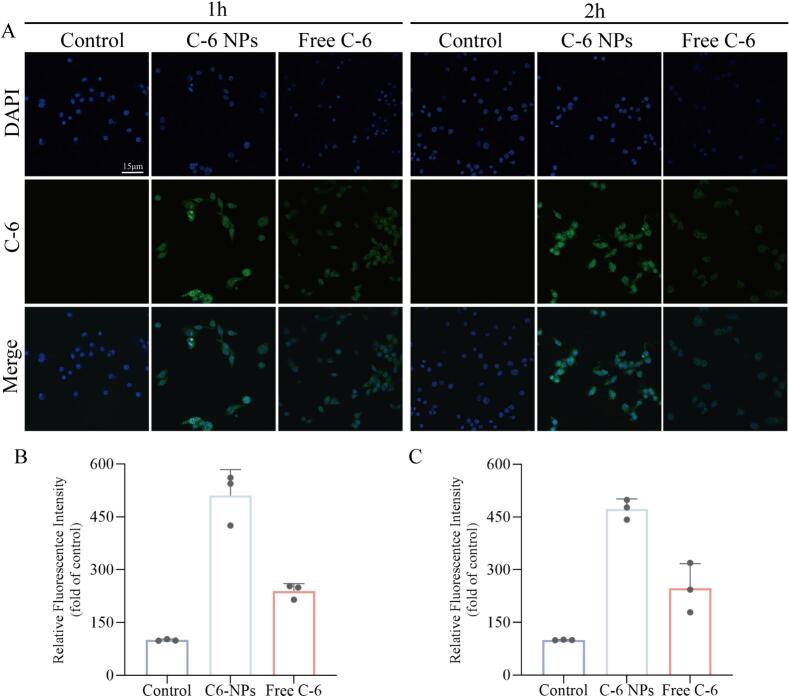
**Uptake of coumarin-6 by MLE-12 cells infected with H1N1 virus.** A. Fluorescence detection image of coumarin-6 uptake by virus-infected MLE-12 cells; B. Quantitative fluorescence analysis of coumarin-6 in MLE-12 cells at 1 h. C. Quantitative fluorescence analysis of coumarin-6 in MLE-12 cells at 2 h.

Coumarin-6 was used as a fluorescent probe to evaluate the uptake behavior of MLE-12 cells toward nanoparticles before and after folic acid modification. After incubation with coumarin-6-loaded nanoparticles for 1–2 h, green fluorescence was predominantly localized in the cytoplasm of uninfected MLE-12 cells, indicating that the nanoparticles effectively delivered coumarin-6 into the cells and accumulated in the cytoplasm. Compared with the free coumarin-6 group, cells treated with folic acid-modified nanoparticles exhibited the highest fluorescence intensity, demonstrating improved uptake efficiency, as shown in Fig. 2. In contrast, virus-infected MLE-12 cells showed even greater uptake of folic acid-modified nanoparticles (Fig. 3), suggesting that the folic acid-modified nanoparticles possess enhanced chemotactic affinity toward MLE-12 cells in an inflammatory state following viral infection. These findings demonstrate the inflammatory targeting capability of this delivery material at the cellular level.

### 4.3 Targeting of nanoparticles to mouse lung tissue

**Fig. 4:**
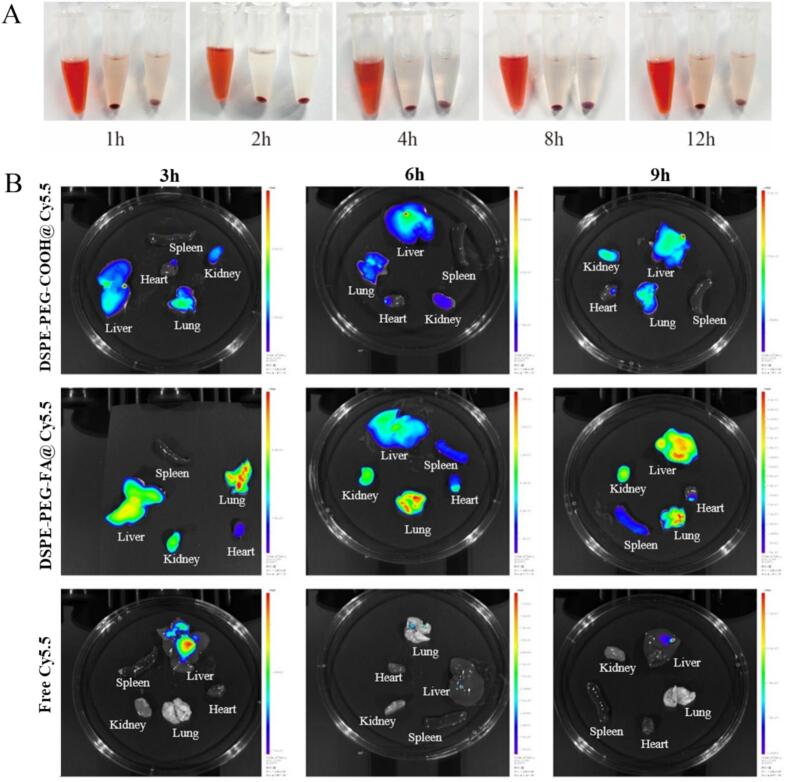
**Targeting of virus-infected lung tissue by folate-modified nanoparticles.** A. Hemolysis test results of MA NPs at different time points. B. Fluorescence distribution and intensity in different organs at each time point.

The biosafety of MA NPs was firstly tested. As shown in Fig. 4A, which presents the hemolysis results at different time points, under various incubation durations, the hemolysis test demonstrated that red blood cells in the Triton X-100 group were completely disrupted, resulting in hemolysis. In contrast, the hemolysis rate of MA NPs was less than 1%, indicating no significant impact on red blood cells. These results suggest that MA NPs possess good biocompatibility and meet the safety requirements for delivery systems.

To evaluate the targeting capability of folic acid-modified nanoparticles to lung tissue in a mouse model of viral pneumonia, mice were intranasally infected with H1N1 virus and then intravenously injected with nanoparticles loaded with the Cy5.5 fluorescent probe.The mice were euthanized at 3, 6, and 9 h post-injection, and the heart, liver, spleen, lungs, and kidneys were collected for ex vivo fluorescence imaging. As shown in Fig. 4B, compared with the free Cy5.5 and non-targeted nanoparticle groups, the folic acid-modified nanocarriers exhibited stronger fluorescence signals in the mouse lung tissue. This result preliminarily demonstrates that folic acid modification significantly enhances the targeted accumulation of nanoparticles in inflamed lungs. In contrast, the accumulation efficiency of free dye and non-targeted nanoparticles in the lungs was relatively limited.

### 4.4 MA NPs reduce the levels of inflammatory cytokines in cells infected with H1N1 virus

**Fig. 5:**
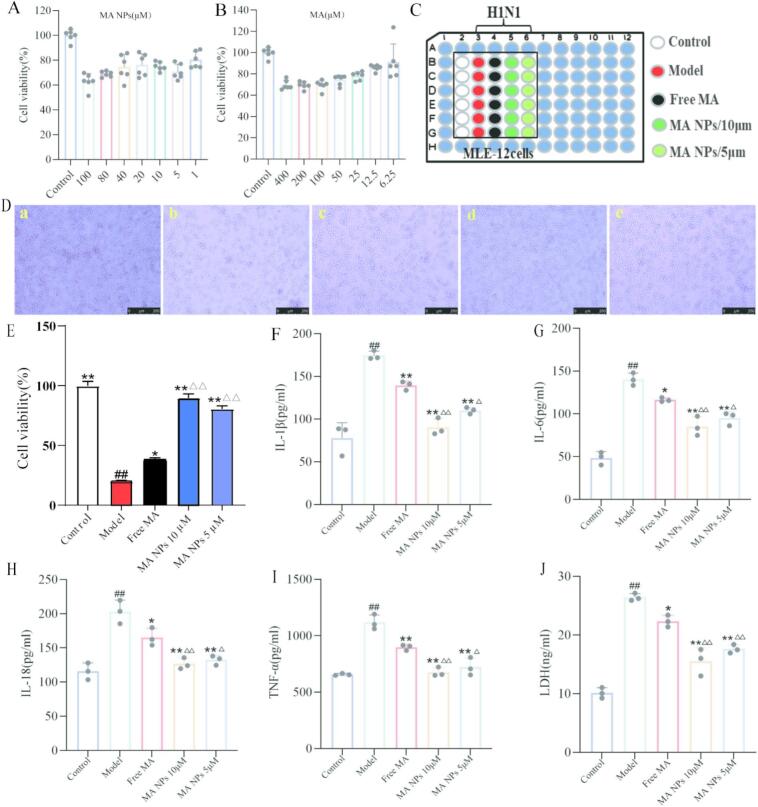
**MA NPs alleviate H1N1 virus-induced inflammation in MLE-12 cells.** (A) Safety evaluation of MA NPs in MLE-12 cells. (B) Safety evaluation of MA in MLE-12 cells. (C) Schematic diagram of experimental grouping and cell treatment. (D) Cytopathic effects in each experimental group after H1N1 virus infection. (E) Cell viability in each group after H1N1 virus infection. (F–G) Levels of IL-1β, IL-6, IL-18, TNF-α, and LDH in cell supernatants of each experimental group. The results represent the mean ± SD of 3 independent experiments. ^##^*P* < 0.01 vs Control group; ^⁎⁎^P < 0.01, ^⁎^*P* < 0.05 vs Model group; ^△△^P < 0.01, ^△^P < 0.05 vs Free MA group.

First, we evaluated the cytotoxicity of free MA and MA NPs in MLE-12 cells. As shown in Fig. 15, free MA did not significantly reduce cell viability at concentrations of 6.25 and 12.5 μM (Fig. 5A). MA NPs also exhibited no obvious cytotoxicity within the concentration range of 1, 5, and 10 μM (Fig. 5B). Experimental grouping was performed as depicted in the schematic diagram in Fig. 5C. At 48 h post H1N1 virus infection, the model group exhibited significant cytopathic effects in MLE-12 cells, as shown in Fig. 5D. In contrast, the MA NPs treatment group markedly alleviated cytopathic effects and significantly increased cell viability, demonstrating excellent protective effects on host cells, as presented in Fig. 5E. The expression levels of IL-1β, IL-6, IL-18, and TNF-α were measured by ELISA in a viral pneumonia model established by H1N1 virus-induced MLE-12 cells following treatment with MA NPs. As shown in Fig. 5F–I, compared with the control group, the expression levels of IL-1β, IL-6, IL-18, and TNF-α were significantly elevated in the model group, indicating successful establishment of the inflammation model and effective activation of the inflammatory response. Following intervention with MA NPs, the levels of these inflammatory factors were markedly reduced, suggesting that MA NPs can effectively suppress the excessive release of inflammatory cytokines in the cellular viral pneumonia model. LDH release reflects increased cell membrane permeability and is a key indicator of pyroptosis. In this study, the level of LDH release in the cell culture supernatant was further assessed to evaluate the extent of pyroptosis. The results showed that LDH activity in the model group was significantly higher than that in the control group (Fig. 5J), indicating that H1N1 virus infection compromised cell membrane integrity and activated the pyroptosis process. Following treatment with MA NPs, LDH release was significantly reduced, suggesting that MA NPs can effectively alleviate the pyroptosis process.

### 4.5 Inhibiting NLRP3-mediated pyroptosis is the primary mechanism by which MA NPs exert their antiviral effect against viral pneumonia

#### 4.5.1 NLRP3 is a key target of MA NPs in the treatment of viral pneumonia

**Fig. 6:**
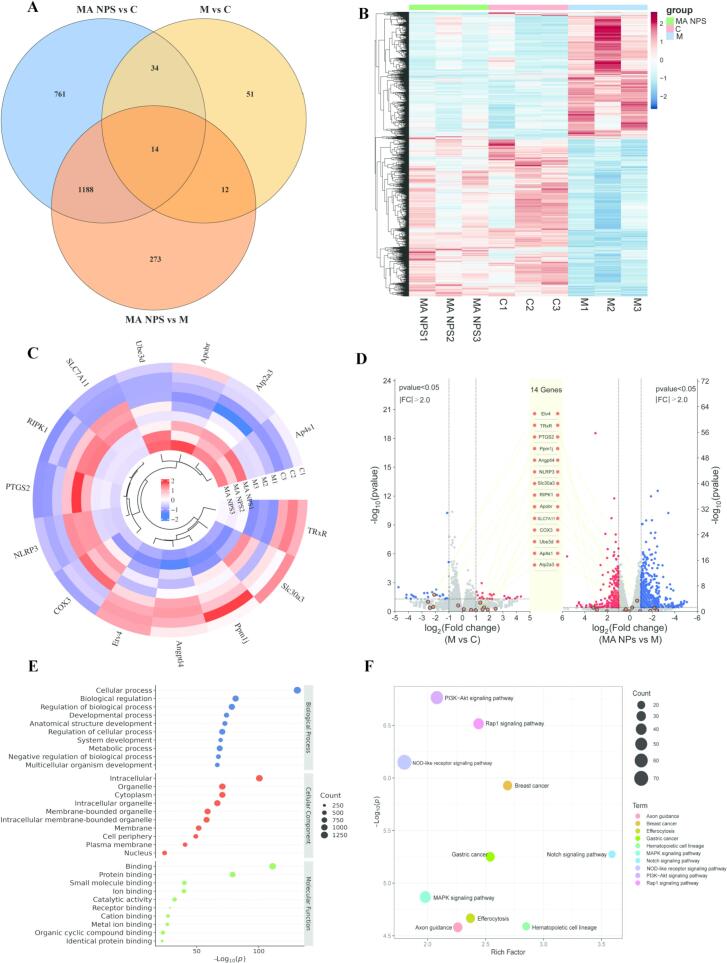
**Comparative analysis of differentially expressed genes across groups.** (A) Venn diagram showing differentially expressed genes and overlapping genes among the three groups. (B) Clustering heatmap showing differences in gene expression trends between groups. (C) Heatmap analysis of 14 differentially expressed genes. (D) Volcano plot of 14 differentially expressed genes. (E) GO functional enrichment analysis of differentially expressed genes. (F) KEGG pathway enrichment analysis of differentially expressed genes.

Venn diagram analysis and heatmap clustering analysis were employed to screen and visualize the differentially expressed genes among the control group (C), model group (M), and MA NPs treatment group. Fig. 6A illustrates the sets of genes with cross-regulation among the three groups, while Fig. 6B visually presents the expression trends of these cross-regulated genes across different treatment groups. Sample clustering analysis based on the differential gene expression profiles showed a clear separation trend among the different treatment groups. Notably, the samples in the MA NPs treatment group clustered more closely with the control group and exhibited significant differentiation from the model group. This finding indicates that after MA NPs treatment, the overall mRNA expression pattern of the cells more closely resembled that of the normal physiological state observed in the control group, while differing substantially from the aberrant expression profile of the model group, preliminarily suggesting that MA NPs may alleviate viral pneumonia by significantly modulating the gene expression profile of MLE-12 cells. Fig. 6C displays the 14 core differentially expressed genes common to all three groups, namely NLRP3, PTGS2, RIPK1, SLC7A11, Ube3d, Apobr, Atp2a3, Ap4s1, TRxR, Slc30a3, Ppm1j, Angptl4, Etv4, and COX3. Further directed expression analysis revealed the specific regulatory patterns of these core genes among the groups. Compared with the control group, the model group showed significant upregulation of five genes—NLRP3, RIPK1, SLC7A11, COX3, and PTGS2—while four genes—TRxR, Ppm1j, Angptl4, and Etv4—exhibited marked downregulation. In stark contrast, as shown in Fig. 6D, compared with the model group, the MA NPs treatment group demonstrated significant downregulation of the five previously upregulated genes and marked upregulation of the four previously downregulated genes, achieving a reverse modulation of the expression patterns.

Fig. 6E presents the GO functional enrichment analysis results of differentially expressed genes. At the cellular component (CC) level, the enriched terms are primarily concentrated in intracellular, organelle, cytoplasm, intracellular organelle, membrane-bound organelle, intracellular membrane-bound organelle, membrane, cell periphery, plasma membrane and nucleus. Among these, intracellular stands out in terms of both the number of enriched genes and statistical significance, indicating that the encoded products of these differentially expressed genes are mainly localized within various intracellular structures, particularly organelles and membrane systems. Fig. 6F presents the KEGG pathway enrichment analysis results of differentially expressed genes. The signaling pathways involved encompass multiple functional categories, including cell proliferation and survival-related pathways such as the PI3K-Akt and Rap1 signaling pathways; immune and inflammation-related pathways such as the NOD-like receptor signaling pathway. Cell fate regulation pathways including ferroptosis and the Notch signaling pathway; stress and cell lineage-related pathways involving oxidative stress and hematopoietic cell lineage; as well as the MAPK signaling pathway, among others. In terms of enrichment characteristics, the NOD-like receptor pathway exhibits the highest -Log₁₀(p) value, indicating strong statistical significance, suggesting that this pathway serves as the core signaling axis through which the differentially expressed genes exert their functions and may be involved in regulating the cellular state and physiological processes within the context of this study.

#### 4.5.2 NOD-like receptor signaling pathway is a key pathway in the treatment of viral pneumonia with MA NPs

**Fig. 7:**
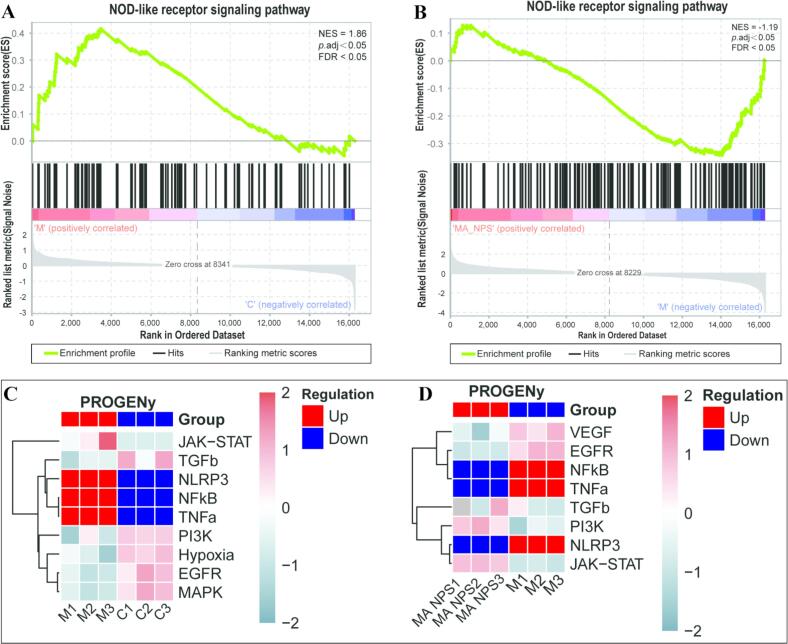
**Regulation of the NOD-like receptor signaling pathway by MA NPs in virus-infected MLE-12 cells.** (A) The NOD-like receptor signaling pathway is overactivated in cells of the model group; (B) The NOD-like receptor signaling pathway is downregulated in cells treated with MA NPs; (C) NLRP3 is upregulated in cells of the model group; (D) NLRP3 is downregulated in cells treated with MA NPs.

To further analyze the expression trend of the NOD-like receptor signaling pathway among the three groups, gene set enrichment analysis (GSEA) was performed to evaluate the expression of this pathway across the groups. Fig. 7 presents an analysis focusing on the regulatory pattern of the NOD-like receptor signaling pathway and associated signaling axes, integrating enrichment curves with PROGENY signaling activity heatmaps to illustrate regulatory changes among the different groups. In Fig. 7 A (model group M vs. control group C), the NOD-like receptor signaling pathway was positively regulated, indicating its activation. In Fig. 7B (MA NPs group vs. model group M), the pathway was negatively regulated, indicating its inhibition.

Correspondingly, the PROGENY heatmap in Fig. 7C shows that genes related to the NOD-like receptor signaling pathway (such as NLRP3, TNF-α, and PI3K) were predominantly upregulated (shown in red) in the model group, suggesting significant positive regulation of this pathway in the model group. The PROGENY heatmap in Fig. 7D reveals that in the MA NPs treatment group, genes such as NLRP3 and TNF-α exhibited downregulation (shown in blue), indicating that after MA NPs treatment, the pathway transitioned from positive regulation in the model group to negative regulation, consistent with the downregulation trend of core genes within the pathway.

In summary, the NOD-like receptor signaling pathway was positively regulated (activated) in the model group (M), accompanied by upregulation of core genes. Following MA NPs treatment, the pathway shifted to negative regulation (inhibition), with corresponding downregulation of pathway-related gene expression, demonstrating the regulatory effect of MA NPs on this inflammation-associated pathway.

#### 4.5.3 MA NPs inhibit the expression of NLRP3, Caspase-1, and ASC-1 in H1N1 virus-induced MLE-12 cells

To elucidate the effect of MA NPs on the NLRP3 inflammasome pathway at the protein level, this study employed Western blotting to detect the expression changes of NLRP3, ASC1, and Caspase1 in a H1N1 virus-induced MLE-12 cell infection model. The results are shown in Fig. 7. Compared with the normal control group, the protein expression levels of NLRP3, ASC1, and Caspase1 were significantly upregulated in the model group, indicating successful activation of the inflammasome. Following intervention with different concentrations of MA NPs, the expression levels of the aforementioned proteins were significantly inhibited. These findings confirm that MA NPs can effectively suppress the assembly of the NLRP3 inflammasome and the subsequent activation of Caspase1, thereby clarifying their therapeutic effect and mechanism in viral pneumonia at the molecular level.

### 4.6 MA NPs inhibit H1N1 virus-induced overexpression of GSDMD

**Fig. 8:**
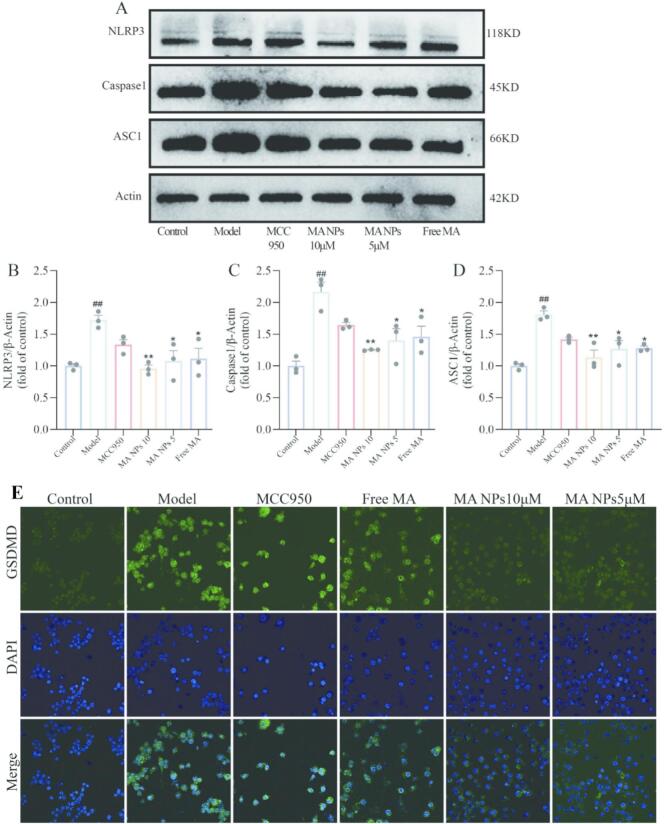
**Effect of MA NPs on the expression of key proteins in the NOD-like receptor signaling pathway.** (A) Representative immunoblots of NLRP3, Caspase-1, and ASC. (B—D) Quantitative analysis of NLRP3, Caspase-1, and ASC protein expression. (E) Immunofluorescence detection of GSDMD in MLE-12 cells. The results represent the mean ± SD of 6 independent experiments. ^##^P < 0.01 vs Control group; ^⁎⁎^P < 0.01, ^⁎^P < 0.05 vs Model group; ^△△^P < 0.01, ^△^P < 0.05 vs Free MA group.

GSDMD is a key execution protein of pyroptosis. In the NLRP3 inflammasome pathway, activated Caspase-1 specifically cleaves GSDMD, exposing its N-terminal pore-forming domain, which inserts into the cell membrane and forms pores, promoting the release of pro-inflammatory factors such as IL-1β and IL-18, ultimately leading to pyroptosis(Wang et al., 2025). In this study, as shown in Fig. 8E, compared with the control group, the immunofluorescence intensity of GSDMD was significantly increased in the model group, indicating that the inflammatory model successfully activated the expression of GSDMD, the key execution protein of pyroptosis. Following intervention with different drugs, all treatment groups exhibited inhibitory effects on the aberrant activation of GSDMD, with the MA NPs group demonstrating the most pronounced inhibitory effect. This suggests that MA NPs possess the potential to regulate the pyroptosis process, and their mechanism of action may be associated with the inhibition of NLRP3 inflammasome activation.

### 4.7 The results of biolayer interferometry (BLI)

**Fig. 9:**
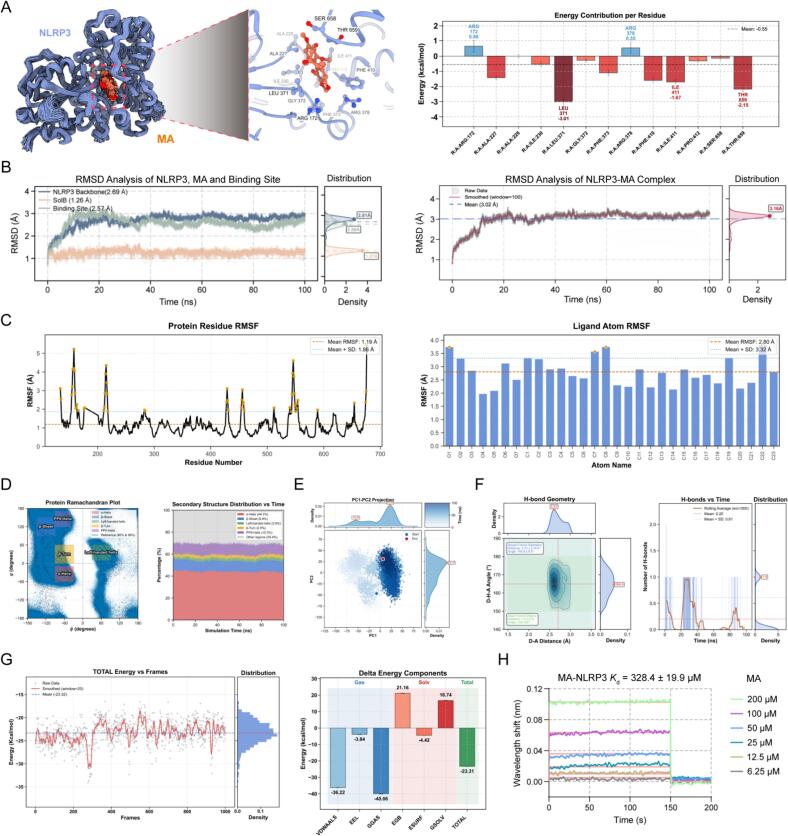
**Molecular Dynamics Simulation and Affinity Analysis of MA and NLRP3.** (A) Molecular docking of NLRP3-MA and residue energy contribution at the binding site. (B) RMSD analysis of the NLRP3-MA complex. (C) RMSF analysis of the NLRP3-MA complex. (D) Analysis of NLRP3 protein conformation: Ramachandran plot and secondary structure distribution over time. (E) PCA analysis of the NLRP3-MA complex. (F) Hydrogen bond interactions and binding free energy analysis of the NLRP3-MA complex. (G) Binding free energy analysis between MA and NLRP3. (H) Affinity constant analysis between MA and NLRP3.

To investigate the potential mechanism by which MA ameliorates viral pneumonia, we first employed molecular docking to study the interaction potential between MA and NLRP3. In this molecular docking study, as shown in Fig. 9A, the MA molecule binds to the active region within the complex three-dimensional folded structure of the NLRP3 protein. MA interacts with multiple amino acid residues of NLRP3 (such as ALA 227, ALA 32, ILE 230, LEU 371, etc.), which contribute differently (marked in red and blue) to the binding with MA. As shown in Fig. 9B, the average RMSD of the NLRP3 protein backbone was 2.69 Å, the average RMSD of the binding site was 2.57 Å, and the average RMSD of MA was the lowest at 1.26 Å. The RMSD curves of all three components remained generally stable during the simulation (0–100 ns), indicating good structural stability of the system. The distribution plots on the right characterize the distribution density of the RMSD for each component, further corroborating the stable state of the structure.

In Fig. 9C, “Protein Residue RMSF” shows that the average RMSF of NLRP3 residues was 1.19 Å. Most residues exhibited low RMSF values with small fluctuations, with only a few residues showing distinct peaks, indicating that the overall conformation of NLRP3 was relatively stable, with only local regions exhibiting certain flexibility. “Ligand Atom RMSF” shows that the average RMSF of MA atoms was 2.80 Å. Although the RMSF values varied among atoms, they remained within a relatively stable range overall, indicating good conformational stability of MA when binding to NLRP3.

Fig. 9D shows that the distribution of φ and ψ angles of the NLRP3 protein was mainly concentrated in typical secondary structure regions such as α-helices, β-sheets, and β-turns, and the proportions of various secondary structures remained relatively stable, indicating high structural stability of NLRP3. In Fig. 9E, the PCA of the NLRP3-MA complex showed distinct distribution and migration characteristics of conformational points along the PC1 and PC2 dimensions. From the beginning to the end of the simulation, significant spatial sampling of conformational states occurred, further supporting the dynamic stability of their interaction.

In the hydrogen bond analysis shown in Fig. 9F, the D-A distance was 2.61 Å, and the D-H-A angle was 164.9 ± 6.8°, both meeting the criteria for ideal hydrogen bonds, indicating that the geometric characteristics of the hydrogen bonds between the two were reasonable. The average number of hydrogen bonds was 0.20, and although it fluctuated over time, the overall distribution had an average of 1.0, indicating a relatively stable hydrogen bond interaction between NLRP3 and MA, providing important intermolecular force support for their binding.

The binding free energy analysis in Fig. 9G indicated that the total energy remained stable throughout the simulation, and its distribution characteristics further supported the consistency of the energy state, suggesting that the energy state of the NLRP3-MA complex was stable. The contributions were as follows: VDWAALS contributed −36.22 kcal/mol, EEL contributed −3.84 kcal/mol, GGAS was −40.06 kcal/mol, EGB was 21.16 kcal/mol, ESURF was −4.42 kcal/mol, and GSOLV was 16.74 kcal/mol. The final total binding free energy (TOTAL) was −23.31 kcal/mol.Subsequently, the interaction between NLRP3 and MA was validated using BLI experiments. The results in Fig. 9H showed a clear direct interaction, with a binding affinity constant of 328.4 ± 19.9 μM determined through kinetic detection.

These results support the feasibility of MA as a potential NLRP3 inhibitor from a structural perspective, providing a reliable basis for subsequent activity validation.

### 4.8 MA NPs exhibit a significant therapeutic effect on viral pneumonia in mice

**Fig. 10:**
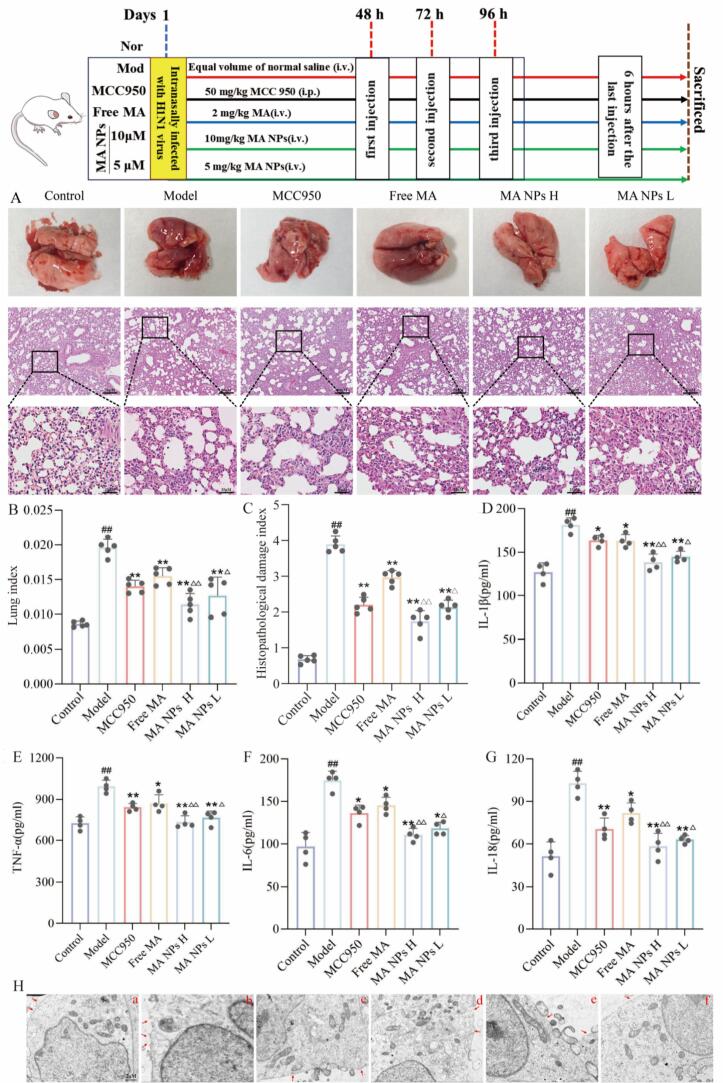
**Therapeutic effect of MA NPs on viral pneumonia in mice.** (A) Pathological morphology of mouse lung tissue. (B) Lung index of mice. (C) Histopathological damage index of mice. (D–G) Inflammatory cytokine levels in lung tissue. (H) Transmission electron microscopy detection of mouse lung tissue. (a) Control group. (b) Model group. (c)MCC950 group. (d) Free MA group. (e) High dose group of MA NPs (MA NPs H). (f) Low dose group of MA NPs(MA NPs L). The results represent the mean ± SD of 5 independent experiments. ^##^P < 0.01 vs Control group; ^⁎⁎^P < 0.01, ^⁎^P < 0.05 vs Model group. ^△^P < 0.05 vs Free MA group; ^△△^P < 0.01 vs Free MA group.

As shown in Fig. 10.A (H&E staining), the lung tissue of the control group exhibited intact structure, clear alveolar spaces, and no interstitial thickening or inflammatory cell infiltration. In contrast, the model group showed severe destruction of alveolar structures, significant thickening of the interstitium, and infiltration of neutrophils and macrophages, indicating successful establishment of the model. Following intervention with MA NPs, the pathological damage to lung tissue in the virus-infected group was markedly alleviated. As a comprehensive functional indicator for assessing lung tissue edema and injury, the lung index (Fig. 10B) and histopathological damage index(Fig. 10C) were significantly increased in the model group, consistent with the inflammatory exudation and edema observed histopathologically. Treatment with MA NPs reduced the lung index, with an effect superior to that of free MA, indicating that MA NPs more effectively alleviate pulmonary edema and improve lung tissue barrier function.

In terms of inflammatory levels, ELISA results (Fig. 10 D-G) showed that the levels of pro-inflammatory cytokines IL-1β, TNF-α, IL-6, and the pyroptosis-related factor IL-18 in the lung tissue of the model group were significantly elevated compared with the control group. After treatment with MA NPs, the levels of these inflammatory factors were effectively suppressed. Moreover, the anti-inflammatory effect of MA NPs was significantly superior to that of free MA, suggesting that the nano-delivery system may enhance efficacy by improving bioavailability or targeted accumulation.

To elucidate the ultrastructural changes in lung tissue cells of mice with viral pneumonia, transmission electron microscopy was employed for observation. The results (Fig. 10G) showed that cells in the control group exhibited intact structure, smooth and continuous plasma membranes, clearly defined intracellular organelles, and no obvious pathological changes. In contrast, cells in the model group displayed typical morphological features of pyroptosis: loss of plasma membrane integrity, with numerous pores of varying sizes or bubble-like protrusions observed on the surface. These changes are consistent with the characteristic manifestations of pyroptosis mediated by GSDMD, including membrane perforation and disruption of intracellular homeostasis. Following intervention with MA NPs, ultrastructural damage was significantly ameliorated. In the treatment group, the integrity of the plasma membrane was largely restored, perforation was markedly reduced, and the structure of intracellular organelles approached normalcy. Compared with the free MA treatment group, the MA NPs group exhibited more pronounced effects in maintaining membrane integrity and reducing organelle damage, suggesting that the nanoformulation may more effectively inhibit the execution of pyroptosis by enhancing targeting or cellular uptake efficiency, providing direct morphological evidence at the ultrastructural level for the superior efficacy of MA NPs.

In summary, this study demonstrates from four aspects—histopathological morphology, inflammatory mediator release, organ function indicators, and cellular ultrastructure—that MA NPs effectively alleviate the pathological progression of viral pneumonia by significantly inhibiting the release of pro-inflammatory cytokines and reducing inflammatory cell infiltration and tissue edema. Moreover, the nano-delivery formulation exhibits superior efficacy compared with the conventional free drug.

### 4.9 MA NPs inhibit the expression levels of NLRP3, GSDMD, Caspase-1, and ASC in mouse lung tissue

**Fig. 11:**
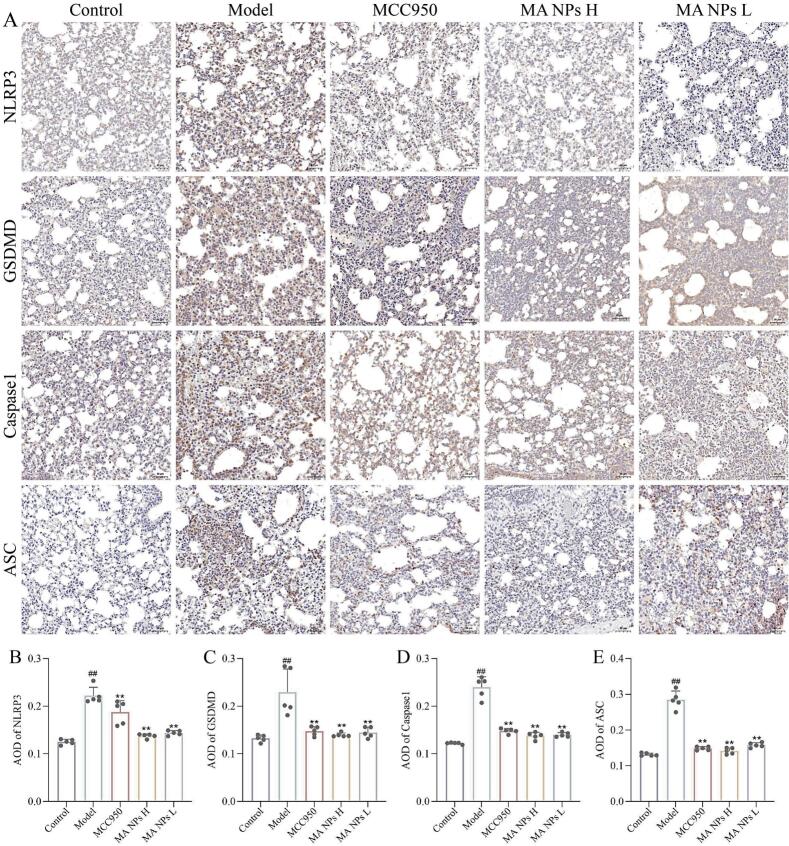
**Effects of MA NPs on NLRP3, GSDMD, Caspase-1, and ASC in lung tissues of mice with viral pneumonia.** (A) Immunohistochemistry (IHC) detection of mouse lung tissues. (B–E) Quantitative analysis of IHC detection. The results represent the mean ± SD of 5 independent experiments. ^##^P < 0.01 vs Control group; ^⁎⁎^P < 0.01, ^⁎^P < 0.05 vs Model group.

Through immunohistochemical staining of lung tissue sections, this study visually characterized the expression and distribution of key proteins of the NLRP3 inflammasome pathway in a viral pneumonia model. As shown in Fig. 11A, compared with the control group, intense brownish-yellow positive signals were observed within the lung tissue structure of the model group, indicating that the expression of NLRP3, ASC, Caspase-1, and GSDMD proteins was significantly induced. These positive signals were primarily localized in the cytoplasm of alveolar epithelial cells, infiltrating inflammatory cells, and vascular endothelial cells, with their diffuse distribution highly coinciding with areas of tissue damage. Morphologically, this confirmed the comprehensive activation of the NLRP3 inflammasome pathway and the subsequent occurrence of pyroptosis in the viral pneumonia model. Following intervention with MA NPs, the intensity and extent of immunopositive reactions for each protein were markedly reduced, suggesting that this treatment effectively inhibits the excessive activation of this pathway.

To further quantitatively evaluate these changes, average optical density (AOD) analysis was performed on the immunohistochemical sections. As shown in Fig. 11 B-E, compared with the control group, the AOD values of NLRP3, ASC, Caspase-1, and GSDMD in the lung tissue of the model group were significantly increased, confirming the overexpression of pyroptosis-related proteins at a semi-quantitative level. Following treatment with MA NPs, the AOD values of all target proteins were significantly reversed compared with the model group. The morphological observations and quantitative data were highly consistent, collectively demonstrating that MA NPs can inhibit the NLRP3-mediated pyroptosis signaling pathway at the protein expression level by specifically downregulating the expression of core components of the NLRP3 inflammasome (NLRP3, ASC, Caspase-1) and its downstream execution protein GSDMD. This may represent one of the key mechanisms by which MA NPs alleviate tissue damage in viral pneumonia.

### 4.10 MA NPs regulate macrophage polarization

**Fig. 12:**
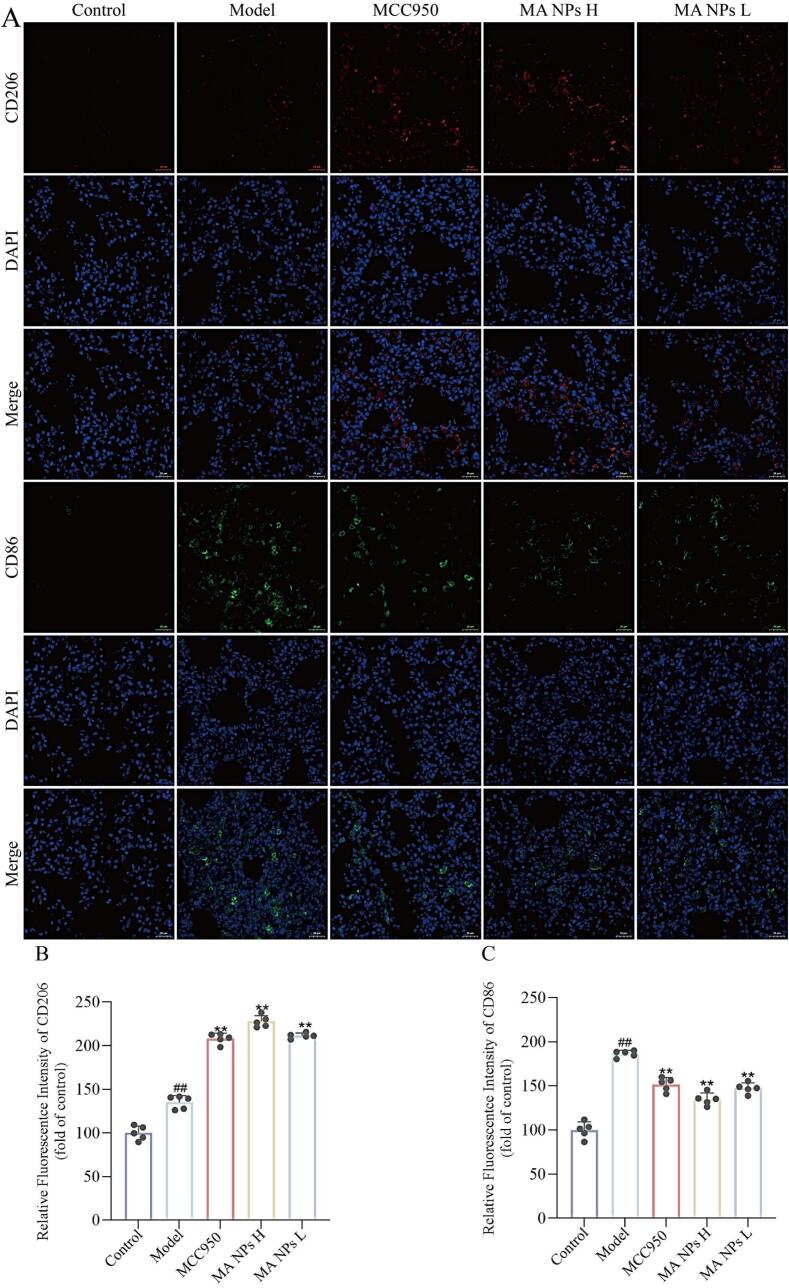
**Effects of MA NPs on CD86 and CD206 in lung tissues of mice with viral pneumonia.** (A) Immunofluorescence detection of CD86 and CD206. (B) Quantitative analysis of CD206 immunofluorescence detection. (C) Quantitative analysis of CD86 immunofluorescence detection. The results represent the mean ± SD of 5 independent experiments. ^##^P < 0.01 vs Control group; ^⁎⁎^P < 0.01, ^⁎^P < 0.05 vs Model group.

By examining the expression of macrophage phenotype-specific markers CD86 and CD206 in lung tissue, we deeply explored the regulatory effect of MA NPs on the immune microenvironment of viral pneumonia and its potential association with the NLRP3-mediated pyroptosis pathway. The results in Fig. 12 show that compared with the control group, the expression level of CD86 protein in the lung tissue of the ALI model group was significantly upregulated, indicating substantial infiltration and polarization of classically activated M1 macrophages at the site of injury. This process, together with the excessive release of factors such as TNF-α and IL-1β, drove inflammatory damage and tissue edema. In contrast, the expression of CD206, a marker of anti-inflammatory and reparative M2 macrophages, was significantly suppressed in the model group. Following intervention with MA NPs, a significant inhibition of CD86 expression was observed, accompanied by a marked upregulation of CD206 expression. These findings indicate that MA NPs effectively inhibit M1 polarization and promote M2 polarization, thereby shifting the injury environment from a state dominated by inflammatory destruction toward a balanced state characterized by immune regulation and tissue repair.

Notably, this macrophage phenotype switching is mechanistically linked to the previously observed inhibition of the NLRP3 inflammasome pathway. M2 macrophages, along with their secretion of key anti-inflammatory factors such as IL-10 and TGF-β, are known to effectively inhibit the assembly and activation of the NLRP3 inflammasome(Zhang et al., 2025b). Therefore, the promotion of M2 polarization via CD206 upregulation by MA NPs likely constitutes a critical negative feedback regulatory mechanism. This not only directly alleviates inflammation but also suppresses the excessive activation of the NLRP3-mediated pyroptosis pathway upstream, thereby accelerating the termination of the inflammatory vicious cycle and creating a favorable microenvironment for lung tissue repair.

### 4.11 MA NPs exhibited high biosafety throughout the treatment period

**Fig. 13:**
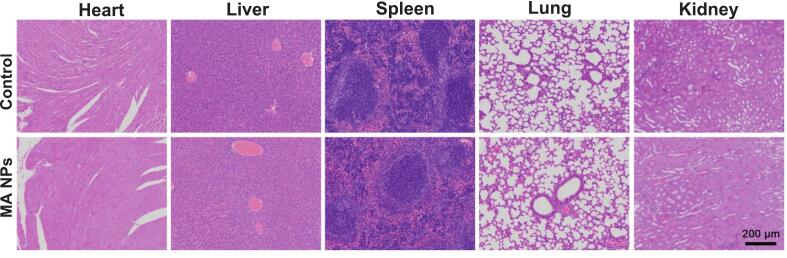
Representative H&E staining images (scale bar = 200 μm) of dissected hearts, livers, spleens, lungs and kidneys of viral pneumonia-bearing mice after 2 weeks of treatment.

After continuous tail vein injection of high-dose MA NPs once daily for two weeks in mice with viral pneumonia, no obvious pathological damage or abnormal immune alterations were observed in major visceral organs, as shown in Fig. 13. These results demonstrate the favorable biosafety of MA NPs throughout the treatment period.

## 5 Discussion

The core pathogenic mechanism of severe pneumonia caused by respiratory viral infections has been confirmed to be cytokine storm, which leads to excessive inflammatory responses, subsequently causing irreversible lung tissue damage, severely affecting patient prognosis and increasing rates of severity and mortality(Gaestel et al., 2021; Karki and Kanneganti, 2021). Therefore, correcting excessive inflammatory responses and restoring immune balance have become the core strategies for treating viral pneumonia, and elucidating the regulatory mechanisms of inflammatory responses is a critical prerequisite for developing effective therapeutic approaches.

Numerous studies have demonstrated that the cytokine storm induced by respiratory viruses is closely associated with excessive activation of inflammasomes. As key initiators and regulators of inflammatory responses, inflammasomes can promote the secretion of IL-1β and IL-18 through Caspase-1, thereby mobilizing immune cells and other cytokines to participate in the regulation of innate and adaptive immunity. However, excessive activation of inflammasomes disrupts immune balance, triggering intense inflammatory responses and ultimately leading to immune-mediated tissue and organ damage. Consequently, the activation mechanisms and homeostatic regulation of inflammasomes have become a focal point in current research on inflammatory responses. Among inflammasomes closely associated with influenza virus infection are the NOD-like receptors, AIM2-like receptors, retinoic acid-inducible gene I, and myxovirus resistance protein A, with the NLRP3 inflammasome being the most active during viral infection(Cabral et al., 2025; Fu et al., 2024; Zhang et al., 2025b).

The NLRP3 inflammasome is composed of NLRP3, apoptosis-associated speck-like protein containing a CARD (ASC), and inactive pro-Caspase-1. Its core function involves recognizing intracellular virus-associated molecular patterns and host-derived danger-associated molecular patterns, mediating the processing, maturation, and release of various cytokines, and directly participating in the initiation and progression of chronic inflammatory diseases(Huang et al., 2021). In the early stages of viral infection; moderate activation of NLRP3 effectively resists viral infection and stress-induced damage; playing a defensive role. However; as the disease progresses; uncontrolled NLRP3 activation amplifies inflammatory effects; leading to organ damage. Clinical studies have found that in patients with severe pneumonia; NLRP3 inflammasome is excessively activated; with abnormally high expression of numerous pro-inflammatory cytokines; triggering cytokine storms and ultimately resulting in lung tissue damage(Li et al., 2025a; Yin et al., 2023). Observations in mouse models infected with influenza A virus also revealed that the expression intensity of NLRP3 inflammasome in the later stages of infection positively correlates with the degree of pulmonary inflammation. In summary, excessive activation of the NLRP3 inflammasome is a critical link in virus-induced cytokine storms and excessive inflammatory responses, representing an important therapeutic target for viral pneumonia.

In-depth investigation into the activation mechanism of the NLRP3 inflammasome reveals that virus-induced NLRP3 activation primarily triggers inflammatory damage through two pathways: first, by mediating Caspase-1 to promote the maturation and secretion of IL-1β and IL-18, directly inducing tissue inflammation, while simultaneously recruiting and activating immune cells such as macrophages, T cells, B cells, and NK cells through these two cytokines, producing large quantities of cytokines involved in immune responses and further amplifying the inflammatory effect(Huang et al., 2021). Second, by mediating Caspase-1 cleavage of Gasdermin D (GSDMD) to induce pyroptosis, leading to the release of intracellular inflammatory substances into the extracellular space and triggering a potent inflammatory cascade reaction. Evidently, abnormal activation of the NLRP3 inflammasome leads to innate immune dysfunction, ultimately resulting in inflammatory pathological damage, which also provides a clear direction for targeted interventions.

In the present study, we demonstrated that MA NPs significantly reduced the expression of NLRP3, ASC, Caspase-1, and GSDMD in MLE-12 cells following H1N1 infection, and also decreased the levels of pyroptosis-related cytokines, suggesting that MA NPs can directly regulate the NLRP3 inflammasome pathway and the pyroptosis process. However, the direct markers of pyroptosis execution—such as cleaved GSDMD—were not measured. Cleaved GSDMD is the direct effector of pyroptotic cell death(Du et al., 2023; Sun et al., 2025), and its absence in the experimental data limits the ability to conclusively establish that MA NPs inhibit the final step of the pyroptosis pathway. Without assessing GSDMD cleavage, it remains uncertain whether the observed reduction in total GSDMD expression translates into a functional blockade of pore formation and subsequent cell lysis. Future studies should therefore include quantification of cleaved GSDMD to provide more definitive evidence that MA NPs directly suppress the pyroptotic execution machinery. Addressing this gap would strengthen the mechanistic understanding of how MA NPs exert their therapeutic effects in viral pneumonia.

To further verify the direct interaction between the drug and the target, molecular docking simulations and bio-layer interferometry (BLI) experimental results indicated that MA molecules could stably bind to the active region of the NLRP3 protein, with a specific direct interaction between them and a binding affinity constant of 328.4 ± 19.9 μM, confirming the potential value of MA as an NLRP3 inhibitor at the molecular structural level.

Mouse model experiments further validated the therapeutic effect of MA NPs on viral pneumonia. Compared with the model group, the lung index in the MA NPs treatment group was significantly reduced, indicating that MA NPs effectively alleviated viral pneumonia-associated pulmonary edema and maintained the integrity of the alveolar-capillary barrier. Simultaneously, the expression levels of pro-inflammatory factors such as IL-1β, TNF-α, IL-6, and IL-18 in the lung tissue of the MA NPs treatment group were significantly downregulated, confirming its significant anti-inflammatory activity. Histopathological observations revealed that the normal control group exhibited intact alveolar structures with thin and uniform alveolar walls. The model group displayed typical pathological features of pneumonia, including thickened alveolar walls, substantial inflammatory cell infiltration within alveolar spaces, and protein exudation. Following MA NPs treatment, these pathological damages were significantly alleviated. Transmission electron microscopy observations further confirmed the protective effect of MA NPs at the ultrastructural level: the control group showed intact alveolar epithelial cells with smooth, continuous cell membranes and clearly defined organelles. The model group exhibited numerous membrane pores or vesicular protrusions of varying sizes on the cell surface. Following MA NPs intervention, ultrastructural damage was significantly ameliorated, and membrane integrity was restored. These results collectively demonstrate that MA NPs exert a significant reparative effect on H1N1 virus-induced lung tissue injury.

To further elucidate the mechanism of action of MA NPs, this study employed immunohistochemistry and immunofluorescence techniques to detect the expression of NLRP3 inflammasome-related proteins (NLRP3, ASC, Caspase-1, GSDMD) and M1/M2 macrophage phenotype markers (CD86, CD206) in lung tissue. The results showed that compared with the control group, the immunopositive signals for NLRP3, ASC, Caspase-1, and GSDMD were significantly enhanced in the model group, indicating successful activation of the NLRP3 inflammasome pathway by H1N1 virus. Following MA NPs treatment, the positive signals for all these proteins were significantly weakened, suggesting that MA NPs effectively inhibit the excessive activation of the NLRP3 inflammasome. Further analysis of macrophage polarization status revealed that the expression of the M1 macrophage marker CD86 was significantly upregulated in the model group. In contrast, after MA NPs treatment, CD86 expression was significantly inhibited, while the expression of the M2 macrophage marker CD206 was notably upregulated. These results indicate that MA NPs not only directly inhibit the NLRP3 inflammasome pathway but also promote the polarization of macrophages from the pro-inflammatory M1 phenotype to the anti-inflammatory/repair M2 phenotype by regulating macrophage polarization, thereby alleviating the pulmonary inflammatory microenvironment, promoting lung tissue repair, and achieving multi-pathway anti-inflammatory and injury-limiting effects.

A core highlight of this study lies in leveraging the specific changes in the inflammatory microenvironment of lung tissue post-viral infection to achieve targeted delivery of MA. The core value of folic acid-modified nanodelivery materials in targeted therapy for viral pneumonia lies in their ability to rely on the high expression of folate receptor beta (FR-β) on the surface of activated inflammatory cells such as macrophages and epithelial cells within pneumonia lesions, enabling the precise accumulation of MA in the pulmonary inflammatory microenvironment through folic acid-mediated active targeting. Compared with traditional administration methods, this strategy not only significantly increases the local concentration of the drug at the lesion site, enhancing the therapeutic effects of inhibiting viral replication and modulating excessive immune activation, but also effectively reduces drug accumulation in non-target organs such as the liver and spleen, minimizing systemic toxicity and side effects. This provides a safe and efficient precision intervention approach to overcome the bottleneck of insufficient efficacy and significant side effects in the treatment of viral pneumonia.

In comparison with previously reported NLRP3-targeted nanotherapies for viral pneumonia, the present work demonstrates several distinct and advantageous characteristics in material design and therapeutic mechanism. Differing from widely used synthetic NLRP3 inhibitors with obvious biosafety risks, Moringa A, a natural plant-derived bioactive component, possesses intrinsic antiviral activity and favorable biocompatibility, which greatly reduces the toxicity concerns of traditional chemical drugs. Moreover, distinct from passive targeting strategies adopted in most existing nanoformulations, folic acid modification endows the prepared nanoparticles with active targeting capability toward folate receptor-overexpressed inflammatory macrophages and alveolar epithelial cells, thereby improving lung tissue accumulation, enhancing local NLRP3 inflammasome inhibition and reducing off-target toxicity. More importantly, different from the single anti-inflammatory modality of conventional NLRP3-targeted nanomedicines, this FA-modified nanosystem achieves a synergistic combination of direct antiviral infection and targeted NLRP3 inflammasome blockade. The dual-mode therapeutic strategy simultaneously inhibits viral proliferation and alleviates NLRP3-driven pulmonary inflammation and pyroptosis, which provides a more effective and actionable solution for the comprehensive treatment of viral pneumonia. Collectively, this natural bioactive molecule-based targeted nanoplatform complements the drawbacks of existing NLRP3-targeted therapies and offers a promising candidate for the clinical translation of viral pneumonia intervention.

In summary, through cellular experiments, animal model validation, and mechanistic investigation, this study demonstrates that MA NPs can mitigate excessive pulmonary inflammation and repair lung tissue damage via suppressing aberrant NLRP3 inflammasome activation and modulating macrophage polarization. Meanwhile, the folic acid-mediated targeted delivery system facilitates targeted drug accumulation in lesion sites, which helps improve therapeutic outcomes and reduce off-target side effects. This work highlights a promising synergistic intervention strategy combining nanocarrier delivery and inflammatory pyroptosis pathway regulation, which shows potential for the targeted intervention of viral pneumonia. In addition, our findings offer preliminary experimental evidence for further in-depth pharmacological research, safety evaluation, and subsequent translational exploration of related therapeutic agents.

## CRediT authorship contribution statement

**Taoyuan Zeng:** Writing – original draft, Software, Investigation, Data curation. **Jiayu Li:** Software, Resources, Investigation, Data curation. **Wenyi Yao:** Validation, Resources, Investigation. **Xu Cheng:** Validation, Software. **Dandan Yang:** Software, Methodology, Formal analysis. **Chunmei Lv:** Validation, Methodology. **Yongai Xiong:** Writing – review & editing, Writing – original draft, Software, Investigation, Funding acquisition.

## Funding declaration

This study was financially supported by the 10.13039/501100001809National Natural Science Foundation of China (No. 82260723).

## Declaration of competing interest

The authors declare that they have no known competing financial interests or personal relationships that could have appeared to influence the work reported in this paper.

## Data Availability

Data will be made available on request.
